# Pediatric to adult healthcare transition: A multifaceted issue

**DOI:** 10.1111/dmcn.70313

**Published:** 2026-06-12

**Authors:** Linda E Krach

**Affiliations:** ^1^ Gillette Children's Specialty Healthcare Saint Paul MN USA

## Abstract

**This commentary is on the original article by Sarmiento et al. on pages** 1412–1420 **of this issue.**

We all experience multiple transitions in our lives. One that has become a frequent topic in healthcare is the transition from pediatric to adult systems. The pediatric system typically offers support (including care coordination) that is unusual in the adult system. This distinction is increasingly important as more individuals with congenital and childhood‐onset disabilities are surviving into adulthood.^1^ There is no criterion standard for measuring the readiness for this transition. However, the Transition Readiness Assessment Questionnaire (TRAQ) is recognized as the most scientifically sound tool available.^2^ It can be helpful in both educating patients and families about considerations for transition to adult services, as well as informing the treating providers about the areas to emphasize in their patient/family education.

Sarmiento et al.^3^ address the uniqueness of cerebral palsy (CP), particularly the heterogeneity, that makes assessing readiness challenging. This qualitative study focused on obtaining feedback about the TRAQ from young adults with CP and caregivers of young adults with CP. The TRAQ includes five domains: managing medications, appointment keeping, tracking health issues, talking with providers, and managing daily activities.^4^ Many participants in this study found that the TRAQ was relatively easy to understand and helpful in reminding them of steps necessary for transition, but participants also noted some challenges. One was that there is not a means of indicating the cognitive ability to manage issues and direct others when one does not have the physical function to carry out the activity independently. Another is that there is not a way to acknowledge that some individuals will always require the assistance of another for decision‐making. Participants noted that managing medications and managing daily activities were two areas that were most helpful and appropriate for them in their consideration of transition to adult healthcare systems. Unfortunately, in the newest version of the TRAQ the questions about managing daily activities were excluded as they did not contribute helpful information for the general population,^4^ but are empirically very relevant for those with CP.

In addition to individual and family readiness for transition, other barriers exist. One is that there are few adult healthcare providers who are comfortable working with individuals with congenital and childhood‐onset disabilities, and their practices may lack resources for working with those with functional disabilities. Got Transition (https://www.gottransition.org/six‐core‐elements/integrating‐young‐adults/), a resource for facilitating successful transition from pediatric to adult healthcare, includes a tool directed toward the receiving practitioner to assess their readiness to accept patients. Perhaps it would be appropriate for Sarmiento et al. to consider not only the creation of an appropriate transition assessment tool for those with CP but also a resource for those planning to accept these individuals into their practices to assess their readiness. Items might include such things as; have you ever worked with an individual with CP previously, are you aware of or have consulted with two or more specialists in your community knowledgeable about CP, is your clinic set up physically for individuals who make use of a wheelchair or assistive communication device, etc.?

## Data Availability

Not required.
